# Longitudinal multi-omics analysis identify multi-kingdom microbiome-host interaction dynamics and diagnostic biomarkers of postoperative infection after kidney transplantation

**DOI:** 10.1038/s41522-026-01099-0

**Published:** 2026-07-20

**Authors:** Yun-Xuan Guan, Lu Wang, Ling-Xiang Kong, Yan-Yan Sun, Jin-An Zhou, Xiu-Li Wang, Yong-Jing Ma, Li-Ping Guo, Tu-Run Song, Lan Zhu, Si-Jun Wang, Ya-Hui Huang, Ruo-Lin Tao, Wen-Jun Shang, Wen-Zhi Guo, Hui Wang, Jia-Yin Yang, Lin Zhong, Gang Chen, Jie Zhao, Ning-Ning Liu

**Affiliations:** 1 State Key Laboratory of Systems Medicine for Cancer, Center for Single-Cell Omics, School of Public Health, Shanghai Jiao Tong University School of Medicine, Shanghai, China; 2 Organ Transplantation Center, West China Hospital of Sichuan University, Chengdu, China; 3 Department of Kidney transplantation, Organ Transplantation Center, Tianjin First Central Hospital, Tianjin, China; 4 Institute of Organ Transplantation, Tongji Hospital, Tongji Medical College, Huazhong University of Science and Technology; Key Laboratory of Organ Transplantation, Ministry of Education, Chinese Academy of Medical Sciences; NHC Key Laboratory of Organ Transplantation, Wuhan, China; 5 Department of Hepatobiliary and Pancreatic Surgery, The First Affiliated Hospital of Zhengzhou University, Zhengzhou, China; 6 Department of Kidney Transplantation, The First Affiliated Hospital of Zhengzhou University, Zhengzhou, China; 7 Department of Liver Surgery and Organ Transplantation Center, Shenzhen Third People’s Hospital, Second Affiliated Hospital, Southern University of Science and Technology, Shenzhen, China; 8 Tianjin Key Laboratory of Organ Transplantation, Tianjin First Central Hospital, Tianjin, China

**Keywords:** Diseases, Microbiology

## Abstract

Despite recent progresses in microbiome and infection, the role of multi-kingdom gut microbiome in kidney transplantation (KT) infection remains unexplored. Here we performed a longitudinal and integrative multi-omics analysis of the gut microbiome, fecal metabolome and plasma metabolome in 169 KT recipients across 5 different transplantation centers, comprising discovery and validation cohorts. We observed KT-specific four kingdom microbiome dysbiosis, including bacteria, fungi, archaea and viruses, with the most pronounced shifts in bacterial and fungal communities. Furthermore, we identified 6 infection-associated co-abundance groups (CAGs) composed of 23 bacterial and 3 fungal species, highlighting extensive bacterial-fungal interactions. Interestingly, infection-associated fecal metabolomic pattern F1, enriched in N-acetylputrescine and hydroxyproline, was positively correlated with *Enterococcus*-, *Citrobacter*- and *Lactococcus*-dominated CAGs, as well as the plasma metabolite signature, represented by phenylacetyl-l-glutamine, indoxyl sulfate and leukotriene. In contrast, cholesterol sulfate and menadione in plasma were aligned with fecal indoleacetic acid and stachyose, a metabolic signature more characteristic of non-infected recipients. Finally, the combinatorial biomarkers of fungal and bacterial species achieved powerful diagnosis ability of KT infection in an independent validation cohort (area under the receiver operating characteristic curve (AUROC) = 0.80) with the fecal metabolites achieving high accuracy (AUROC = 0.83). Collectively, our findings not only uncovered the postoperative infection-specific multi-kingdom microbial network dynamics, but also revealed the microbial and its metabolic biomarkers with powerful diagnostic ability for postoperative infection in kidney transplantation.

## Introduction

Kidney transplantation (KT) is the effective and definitive therapy for patients with end-stage renal disease (ESRD), which globally impacting over 80 million people^1^. However, the post-KT ‘net state of immunosuppression’ leads to a high incidence of postoperative infections^2,3^, which are aggravated by gut microbiome dysbiosis, resulting in poor prognosis and mortality^4,5^. It was reported that gut microbiome was highly correlated with the high incidence of post-transplant urinary tract infections (UTI), respiratory infections, and bloodstream infections. An increased *Firmicutes*-to-*Bacteroidetes* ratio was observed in the gut microbiome of KT recipients^6–8^. Furthermore, there is an elevated abundance of *Proteobacteria*, which may persist for years, suggesting a stable post-transplant microbiome composition over time^9–11^. The extensive colonization of potential pathogens such as *Enterococcus* and *Enterobacterales*, has been linked with post-transplantation UTI^9,12^. Apart from that, the gut fungal expansion, particularly *C. parapsilosis* domination, precedes *Candida* bloodstream infections in haematopoietic cell transplantation (allo-HCT) patients^13^. The gut microbiome derived metabolites are also predicted to be correlated with postoperative pathogen expansion. During liver transplantation^14^, the primary bile acids, tyramine, and amino acids were identified as risk factors for infection. The treatment with immunosuppressive drugs and prophylactic antibiotics after KT caused a decrease in short-chain fatty acid (SCFAs)-producers while impairing the metabolic potential of the gut flora^15,16^. However, the multi-kingdom gut microbiome and its correlation with postoperative infection in KT recipients remain unexplored.

Here we performed a longitudinal and integrative multi-omics analysis of the gut microbiome, fecal metabolome and plasma metabolome in 169 KT recipients across five different centers. We constructed the multi-kingdom microbial network and identified the bacteria-fungal composed co-abundance groups (CAGs) associated with KT postoperative infection. These infection-specific gut microbiome were highly correlated with host plasma metabolites through fecal metabolites. Most importantly, the diagnostic model based on microbes and metabolites all achieved high accuracy. These findings uncovered postoperative infection-associated multi-kingdom microbiome dysbiosis and revealed its diagnostic potential.

## Results

### Dynamic multi-kingdom microbiome dysbiosis after kidney transplantation

**Fig. 1:**
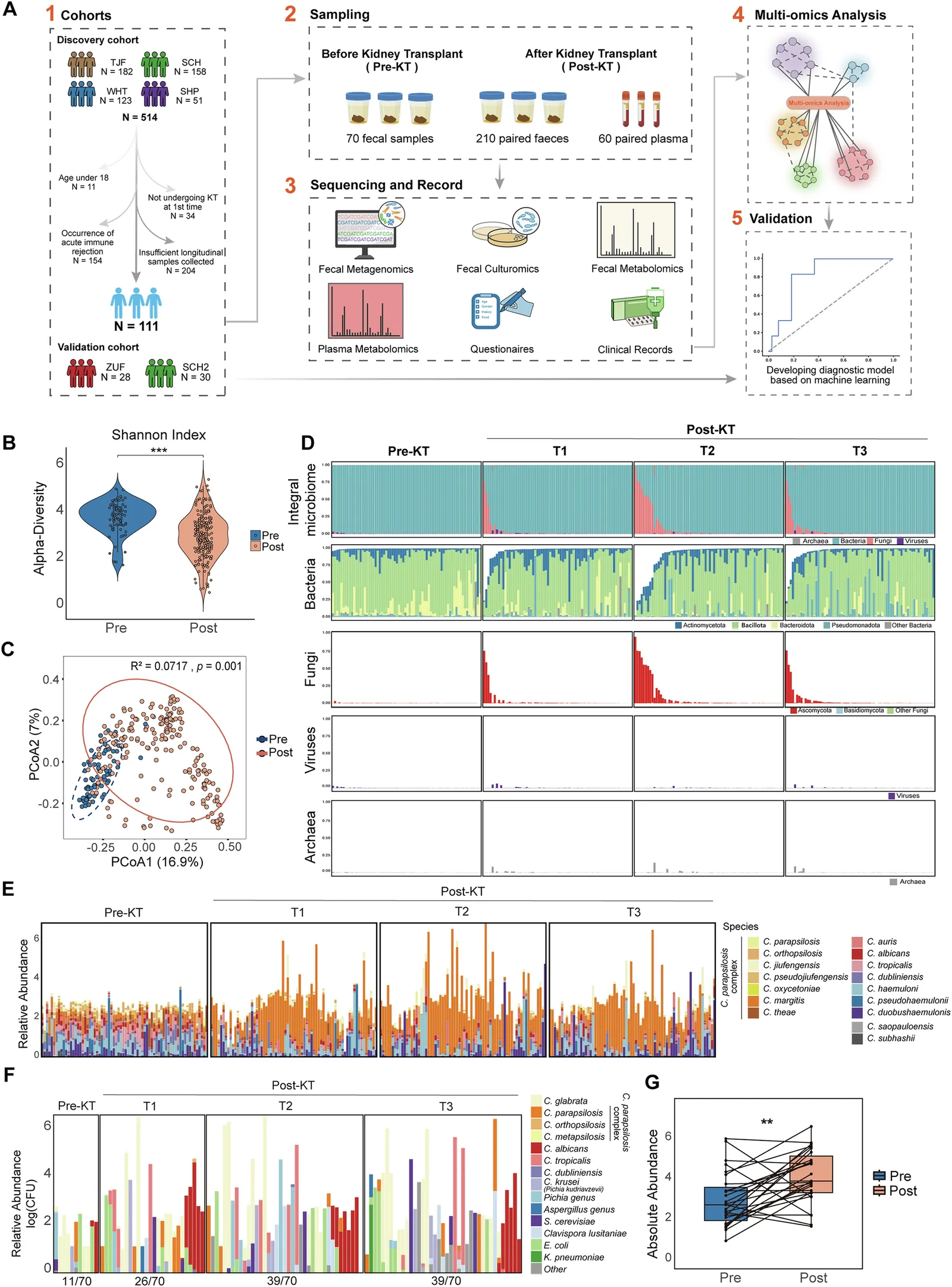
Dynamic dysbiosis of multi-kingdom microbiome after kidney transplantation. **A** Overall schematic representation. **B** Alpha-diversity measured by Shannon index of the microbiome in recipients received kidney transplantation (orange, *n* = 210) and pre-transplantation (blue, *n* = 70). **C** Principal coordinate analysis (PCoA) of samples based on Bray-Curtis distance in recipients received kidney transplantation (orange, *n* = 210) and pre-transplantation (blue, *n* = 70) (*R*^*2*^ = 0.0717, *p* = 0.001). **D** The relative abundance of four microbial kingdoms (Bacteria, Fungi, Viruses, and Archaea) at four stages: Pre-KT, T1, T2 and T3. Top panel: Stacked bar plots showing Integral microbiome abundance. Second panel: bacterial phylum distribution. Third panel: fungal phylum distribution. Forth panel: Viral distribution. Bottom panel: Archaeal distribution. **E** Stacked bar plots displayed the relative abundance of species (as shown in the legend) in *Candida* genera by Metagenomic analysis. Each bar represents an individual sample. **F** Stacked bar plots display the relative abundance of species (as shown in the legend) in *Candida* genera by Culturomic analysis. Each bar represents an individual sample. **G** Comparison of fungal burden in 28 paired pre- and post-transplantation fecal samples quantified by real-time PCR. Data were presented as mean ± SEM. Statistical significance (ns: not significant, *: *p* < 0.05; **: *p* < 0.01; ***: *p* < 0.001) were determined by unpaired two-tailed Student’s *t* test (**B**), permutation multivariate analysis of variance (**C**) and paired two-tailed Student’s *t* test (**G**).

In this study, we performed a longitudinal multi-omics study in 169 kidney transplant recipients recruited from different clinical centers. Among them, 111 recipients constituted the discovery cohort and provided feces and paired plasma samples between day -1 and 30 during transplantation. The samples were collected at Pre-KT (within 3 days prior to transplantation), T1 (the first defecation after operation), T2 (between postoperative day 7–14, during hospitalization) and T3 (the final sample before discharge). These samples were analyzed for gut microbiome, fecal metabolome and plasma metabolome. In addition, an independent validation cohort of 58 KT recipients was established to evaluate the robustness and generalizability of the identified biomarkers (Fig. 1A).

By integrating the four-kingdom microbiome including bacteria, fungi, archaea and viruses, we observed the decreased alpha diversity of (*p* < 0.001) and significantly altered microbial composition by Bray-Curtis distance-based PCoA analysis (*R*^*2*^ = 0.0717, *p* = 0.001) after KT (Figs. 1B, C and S1). Interestingly, an increase in fungal burden accompanied by a reduced bacterial abundance was found after transplantation, especially at T2 (Fig. 1D). Consistent with previous studies^10^, the bacterial community profiling based on relative abundance demonstrated a progressive decline in the *Bacteroidota/Pseudomonadota* ratio over time (Fig. S2A; Kruskal-Wallis, *p* = 3.93 × 10^−14^), dominated by the genera *Enterobacter* and *Citrobacter* within *Pseudomonadota* (Fig. S2B). Within the fungal community, *Candida* exhibited the most pronounced variation (Fig. S2C). We therefore further characterized the species composition of *Candida* by calculating the relative contribution of each *Candida* species within the total fungal community. Notably, the *Candida parapsilosis* complex was significantly enriched post-KT, followed by *Candida albicans* (Fig. 1E). We further validated the presence of *Candida* species by culturing the fecal samples on selective media containing three antibiotics. The *C. parapsilosis complex* and *Candida glabrata* were among the most frequently isolated fungal species (Fig. 1F). This observation is consistent with the gut fungal shifts following allo-HCT^13^. The fungal load after KT was also quantified and confirmed to be increased by qPCR (Fig. 1G). These results thus demonstrated the dynamic four-kingdom microbiome dysbiosis following KT, particularly fungal microbiome.

### The postoperative infection specific co-abundance groups (CAGs)

Postoperative infections remain a major cause of morbidity and mortality following KT and are closely linked to host-microbiome interactions^2^. We did not discover a clear association between the gut microbial changes and specific infectious pathogens (Fig. S3, Table S1). Therefore, rather than focusing on pathogen, we further explored specific microbial signatures potentially predictive of postoperative UTIs in our cohort.

**Fig. 2:**
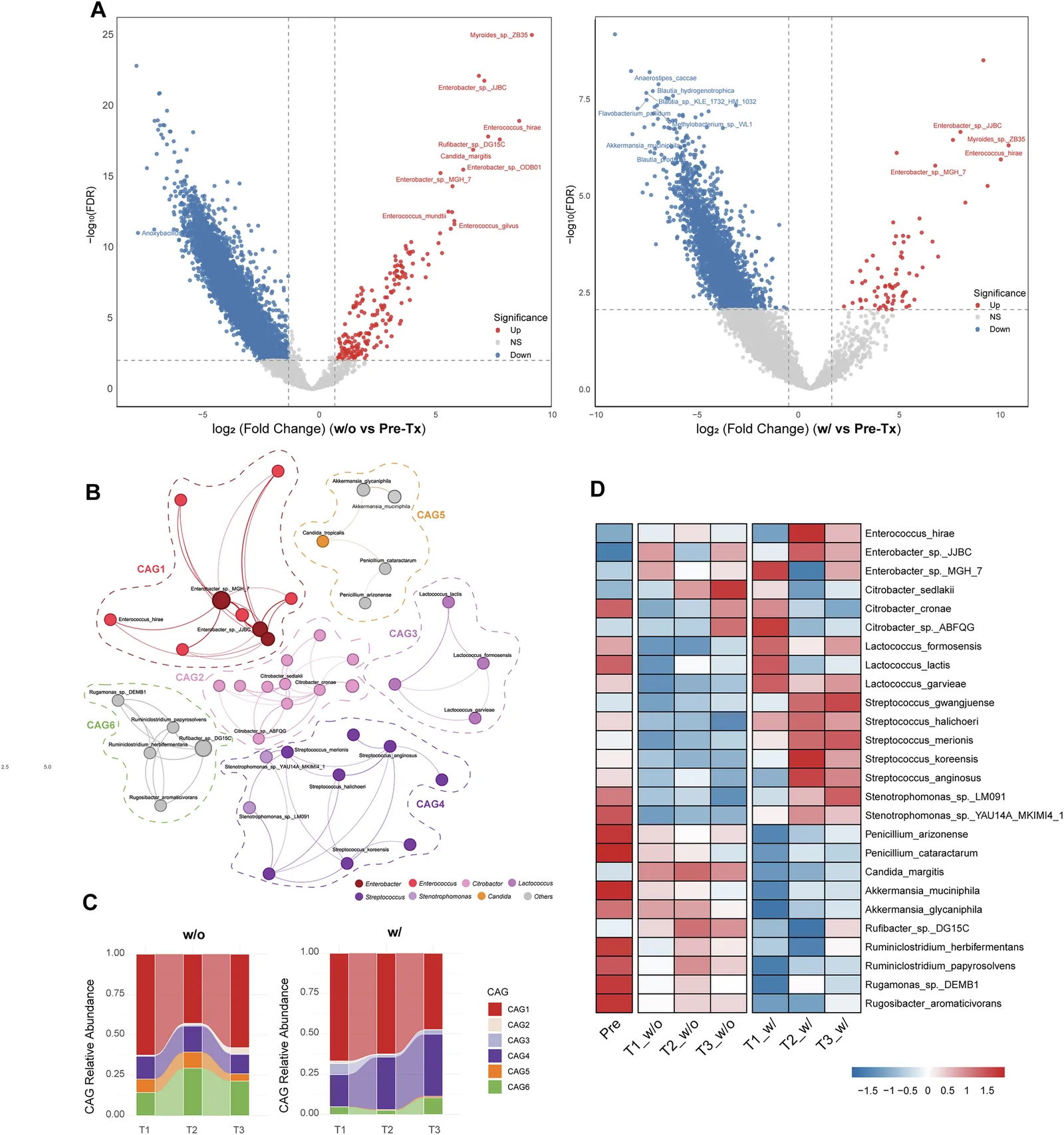
The microbial co-abundance groups associated with postoperative infection. **A** Volcano plot showed differentially abundant microbial species between non-infected recipients (w/o, left) and infected recipients (w/, right) against pre-KT. Each dot represents one microbial species, with the x-axis showing the log_2_(Fold change) and the y-axis showing the −log10(FDR). Vertical and horizontal dashed lines indicate thresholds for significance (|log2FC| ≥ 1 and FDR < 0.1). Differentially enriched species in the infected group are colored in red (Up), while depleted species are in blue (Down); gray points denote non-significant (NS) species. Bacterial and fungal species with highly significant changes are labeled. **B** Co-abundance network was constructed using Spearman correlation coefficients and only significant correlations (*p* < 0.0001, |r| > 0.5) were shown. Overview of the microbial co-abundance groups identified by Louvain algorithm. **C** Alluvial plots displayed the relative abundance of six CAGs in non-infected (left) and infected (right) recipients. **D** The heatmap shows the relative abundance across different time points of dominant microbial species of each CAG. Statistical significance were determined via Wilcoxon test, corrected for multiple testing using FDR. Statistical significance were determined by linear model of MaAsLin2.

Because gut microbial composition can be substantially influenced by host and environmental factors, potential confounders were first assessed. Baseline comparison of recipients with available postoperative samples revealed a sex difference between infected and non-infected groups (Table S2). Although the microbial composition differed across transplantation centers, the effect size was limited (PERMANOVA *R*^2^ < 0.1 for all comparisons) (Fig. S4). After adjustment for confounding factors including center, individual, age and sex, differential species was assessed by Multivariate Association with Linear Models 2 (MaAsLin2) analysis. Based on the postoperative microbial structure, we respectively compared the microbiome of infected and non-infected recipients to the pre-transplant profiles. We found that *Enterobacter sp. JJBC*, *Enterobacter sp. MGH7*, and *Enterococcus hirae* exhibited significant enrichment (log_2_(Fold Change) > 5) in both infected and non-infected recipients (Fig. 2A). Additionally, a total of 208 species were significantly associated with infection (FDR < 0.25), including 98 positively and 110 negatively associated species (Fig. S5A). Given the temporal heterogeneity in microbial dynamics, we further stratified the analysis by different stages after transplantation. We performed the bootstrap-based downsampling at T1, T2, T3 and presented the top 30 bacterial or fungal species (ranked by log_2_(Fold change)). At T1, enrichment of *Lactococcus* sp. was observed, while *Alistipes* and *Akkermansia* were depleted. At T2, *S. gwangjuense*, *S. halichoeri*, and *S. koreensis* were most strongly associated with infection. The abundance patterns were shifted toward *S. halichoeri* and *Corynebacterium propinguum* at T3 (Fig. S5B–D).

The gut microbiome can influence host as a complex micro-ecological system^17^. We thus constructed a co-abundance network using all infection-associated differential microbial species (including those significantly altered postoperatively within the infected recipients and those differentially abundant between infected versus non-infected recipients), and applied Louvain modularity^18^ analysis to delineate the CAGs, which were used to defined community-level clusters based on dominant microbial species (Fig. 2B). We uncovered six CAGs in which CAG1 (*Enterobacter*-dominated) was characterized by *Enterobacter* species and *Enterococcus hirae*. CAG2 and CAG3 were dominated by *Citrobacter* and *Lactococcus* species, respectively, both enriched in infected recipients. For *Streptococcus*-dominated CAG4, it was primarily composed of *Streptococcus sp*. and *Stenotrophomonas sp*. Finally, CAG5 and CAG6 were designated as *Penicillium*- and *Ruminiclostridium*-dominated groups, respectively. To address the potential influence of dietary covariates on the microbial community and infectious status, we explored the correlations between dietary intake, immunosuppressive regimens, and antibiotics exposure with CAGs. Notably, vitamin A intake was consistently correlated with the abundance of *Lactococcus*-dominated CAG3 (Fig. S6).

Next we compared the longitudinal dynamics of distinct CAGs between infected and non-infected recipients. CAG1-4 were consistently enriched in individuals who developed infections, whereas CAG5 and CAG6, which included both bacterial and fungal taxa, were markedly depleted in the infected group (Fig. 2C). To further characterize the ecological features within each CAG, representative microbial species were selected based on log_2_(Fold Change) values in at least one comparison, and their longitudinal abundance patterns were visualized by heatmap. Overall, the representative species within CAG1 showed a generalized postoperative enrichment pattern regardless of infection status. In contrast, species from CAG2 and CAG3 exhibited transient increases predominantly at the early stage of infection. CAG4-associated species displayed persistent enrichment across all postoperative time points in infected recipients, while representative species within CAG5 and CAG6 were consistently depleted in infected recipients after transplantation (Fig. 2D). These findings further support the presence of distinct infection-associated microbial community features characterized by temporally coordinated ecological shifts.

### The altered gut microbiome derived metabolites in infected recipients

**Fig. 3:**
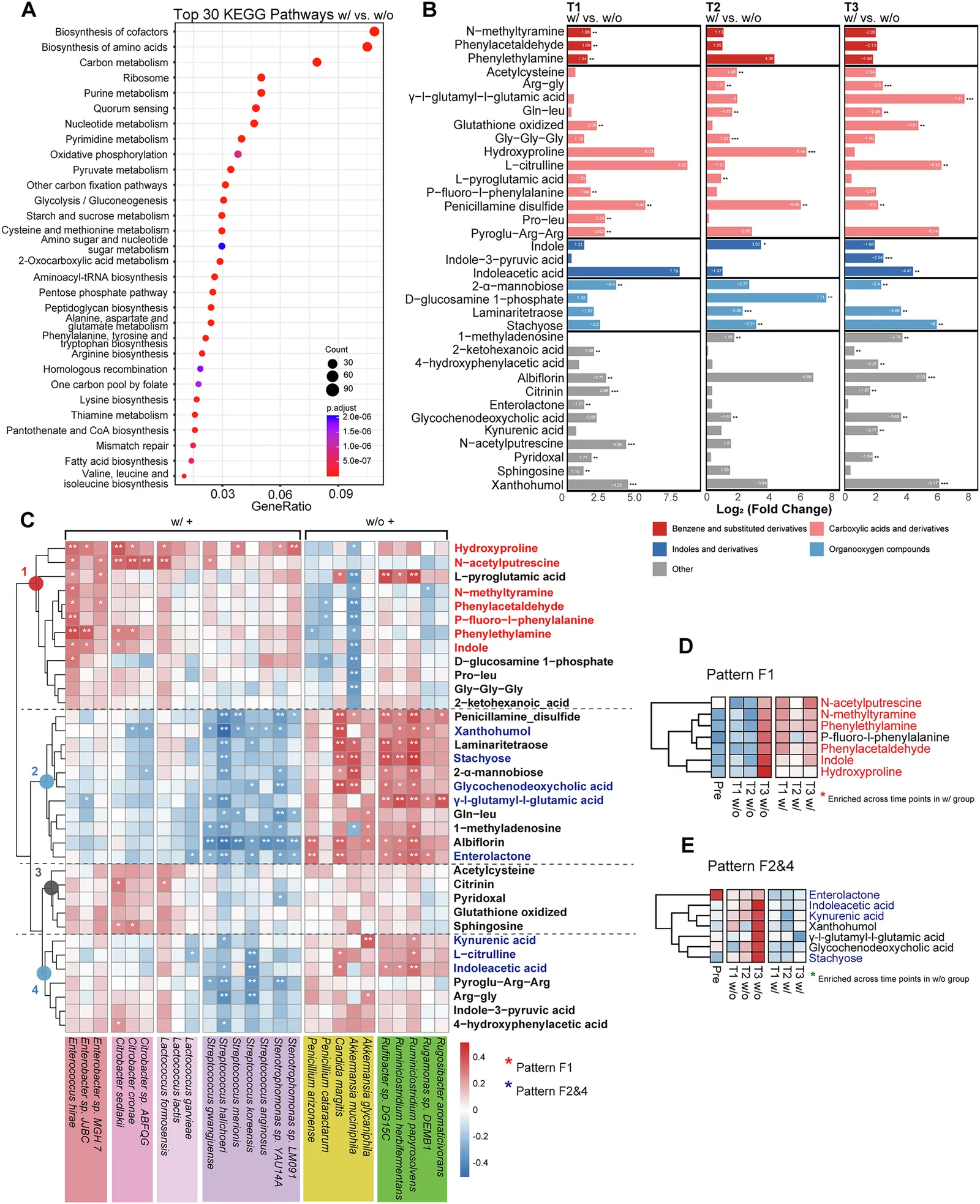
The gut metabolites associated with postoperative infection. **A** KEGG pathway enrichment displays the top 30 enriched KEGG functional pathway of infection compared non-infection group. The color intensity represents the level of enrichment, with the most significantly enriched pathways indicated at the top. **B** The bar plots represent Differential gut metabolites between the infected and non-infected recipients across T1, T2, and T3. The color of each metabolite corresponds to different chemical classes. Log_2_(Fold Change) values and statistical significance were displayed for each metabolite. **C** Correlation heatmap between microbiome and fecal metabolites. Spearman was used to calculate the correlation matrix, with colors representing the strength. Hierarchical clustering was applied to group 4 similar metabolite patterns. The heatmap shows the relative abundance across different time points of putative microbe-derived fecal metabolites in pattern F1, F3 **D**, and pattern F2 **E**. Data were represented as log_2_(Fold Change) (**B**), correlation coefficient (**C**) or mean (**D**, **E**). Statistical significance (ns: not significant, *: *p* < 0.05; **: *p* < 0.01; ***: *p* < 0.001) were determined by non-parametric statistical tests (**B**) and Spearman rank correlation analysis (**C**).

Given the vast inter-individual heterogeneity of the gut microbiome, we hypothesize that distinct species may contribute to similar pathological outcomes by the convergent metabolic activities^19^. We first performed differential abundance analysis of microbial functional genes between infected and non-infected recipients using DESeq2. The significantly altered KO features (adjusted *p* value < 0.05) were subsequently subjected to Kyoto Encyclopedia of Genes and Genomes (KEGG) pathway enrichment analysis. The top enriched pathways included Biosynthesis of cofactors, Carbon metabolism, and Amino acid biosynthesis (Fig. 3A).

Given the significant alterations in metabolism-related pathways and genes, we performed the gut metabolomics analysis by untargeted LC-MS of fecal samples and identified 35 metabolites with significant differences of abundance (related to amino acid, carbohydrate, and purine metabolism) between infected and non-infected recipients (|log_2_(Fold change)| > 1). By classification into distinct metabolic clusters (Fig. 3B), we found that N-acetylputrescine, an intestinal biomarker linked to inflammation and tumorigenesis originated from ornithine, was significantly elevated at T1^20^. In contrast, L-citrulline, an arginine originator indicative of intestinal barrier integrity^21^, was declined at T3 in infected recipients. Tryptophan-derived immunoregulatory metabolites, such as kynurenic acid and indoleacetic acid, were consistently reduced at different time points in infected recipients. We found that elevated levels of Enterolactone was associated with dietary fiber intake (Fig. S7), a gut microbial metabolite known to be derived from dietary lignans^22,23^.

To assess the correlation between gut microbiome and its derived metabolites, we next performed unsupervised hierarchical clustering based on the metabolite-microbe correlation matrix and discovered four distinct metabolite patterns. Two metabolite patterns (F2 and F4) were negatively associated with *Streptococcus*-dominated CAGs and positively associated with CAG5 and CAG6. While Pattern F1 was positively correlated with *Enterococcus*-, *Citrobacter*- and *Lactococcus-*dominated CAGs (Fig. 3C). To understand the biological relevance of these patterns with infection, we annotated gut metabolites using MetaBio and PubChem. Pattern F1 remained persistently elevated in infected recipients and was characterized by metabolites potentially associated with diseases, including N-acetylputrescine^20^, hydroxyproline^24^, and phenylethylamine (Fig. 3D). In contrast, Pattern F2 and F4, which included postbiotics such as enterolactone, stachyose^25^, indoleacetic acid, and kynurenic acid, consistently exhibited lower abundance in infected recipients (Fig. 3E).

### The host metabolism in infected recipients impacted by dysbiotic gut microbiome and its derived metabolites

**Fig. 4:**
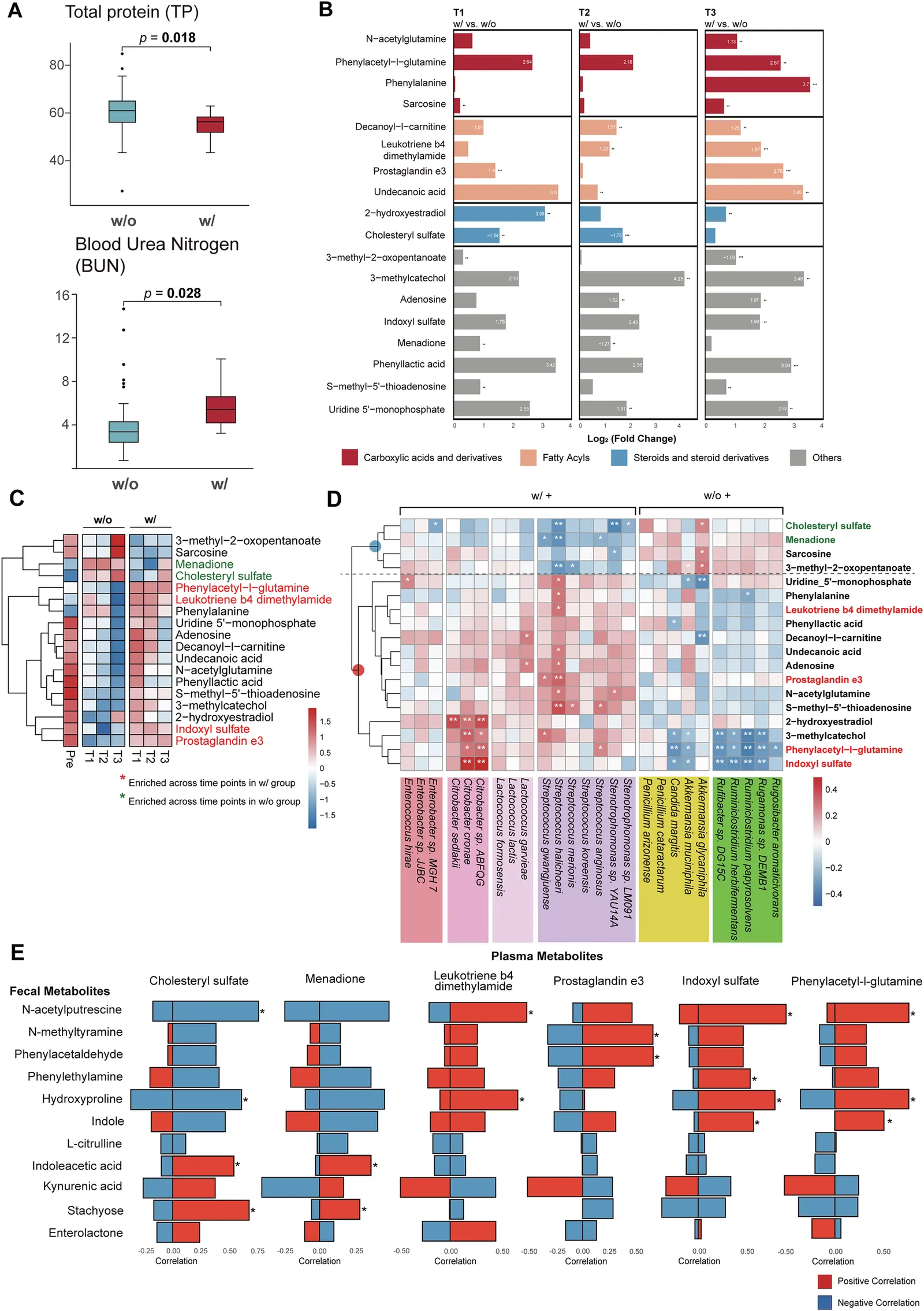
The host metabolism in infected recipients impacted by gut microbiome and its derived metabolites alteration. **A**. Comparison of biochemical indicators in non-infection and infection patients. **B**. The bar plots represent differential plasma metabolites between the infection and non-infection groups across T1, T2 and T3. The color of each metabolite corresponds to different chemical classes. Log_2_(Fold Change) values and statistical significance were displayed for each metabolite. **C**. The heatmap shows the relative abundance across different time points of plasma metabolites. **D**. Correlation heatmap between microbiome and plasma metabolites. Spearman was used to calculate the correlation matrix, with colors representing the strength. Hierarchical clustering was applied to group metabolite patterns. **E**. The horizontal bar plot represent the correlation between putative microbe-derived fecal metabolites and plasma metabolites in non-infection group (left) and infection group (right). Statistical significance were displayed for each metabolite. Data were presented as mean ± SEM (A), Log_2_(Fold Change) (B) or correlation coefficient (D). Statistical significance (ns: not significant, *: *p* < 0.05; **: *p* < 0.01; ***: *p* < 0.001) were determined by unpaired two-tailed Student’s *t* test (A), non-parametric statistical tests (B) and Spearman rank correlation analysis (D).

To assess the host responses between infected and non-infected recipients, we compared the clinically biochemical indicators collected after transplantation (Fig. 4A). The infected recipients exhibited significantly higher blood urea nitrogen (BUN) levels (*p* = 0.028) and lower total protein (TP) levels (*p* = 0.018), consistent with prior observations that hypoalbuminemia may increase infection risk in immunosuppressed host^26^.

We performed untargeted LC-MS analysis of plasma and matched fecal samples from T1 to T3. We identified 18 plasma metabolites that differed significantly between infected and non-infected recipients (|log_2_(Fold change)| > 1 in at least one time point) (Fig. 4B). Several metabolites, including phenylacetyl-l-glutamine, leukotriene B4 dimethylamide, indoxyl sulfate, and prostaglandin E3, exhibited persistent elevation across all three timepoints in infected recipients and were consistently low in non-infected recipients. Conversely, menadione and cholesteryl sulfate were persistently elevated in non-infected recipients but only partially recovered at T3 in infected recipients (Fig. 4C). Notably, we found the correlation between microbial community structures and host metabolites. The phenylacetyl-l-glutamine, leukotriene B4 dimethylamide, indoxyl sulfate, and prostaglandin E3 showed strong positive correlations with the *Streptococcus*-dominated CAG4. However, cholesteryl sulfate and menadione, which were reduced in infected recipients, showed significantly negative correlations with the *Streptococcus*-dominated CAG4 (Fig. 4D). These results suggest that plasma metabolite alterations in infected recipients are associated with specific gut microbial community structures.

Furthermore, we found more positive associations between gut metabolites and plasma metabolites in the infected recipients (Fig. S8). Among the differential metabolites associated with infection, fecal N-acetylputrescine, hydroxyproline and indole were positively correlated with plasma phenylacetyl-L-glutamine, leukotriene B4 dimethylamide, and indoxyl sulfate in infected recipients, whereas plasma cholesterol sulfate and menadione showed coordinated variation with fecal indoleacetic acid and stachyose (Fig. 4E).

### The diagnostic models of postoperative infection based on the multi-kingdom microbial marker and metabolite panels

**Fig. 5:**
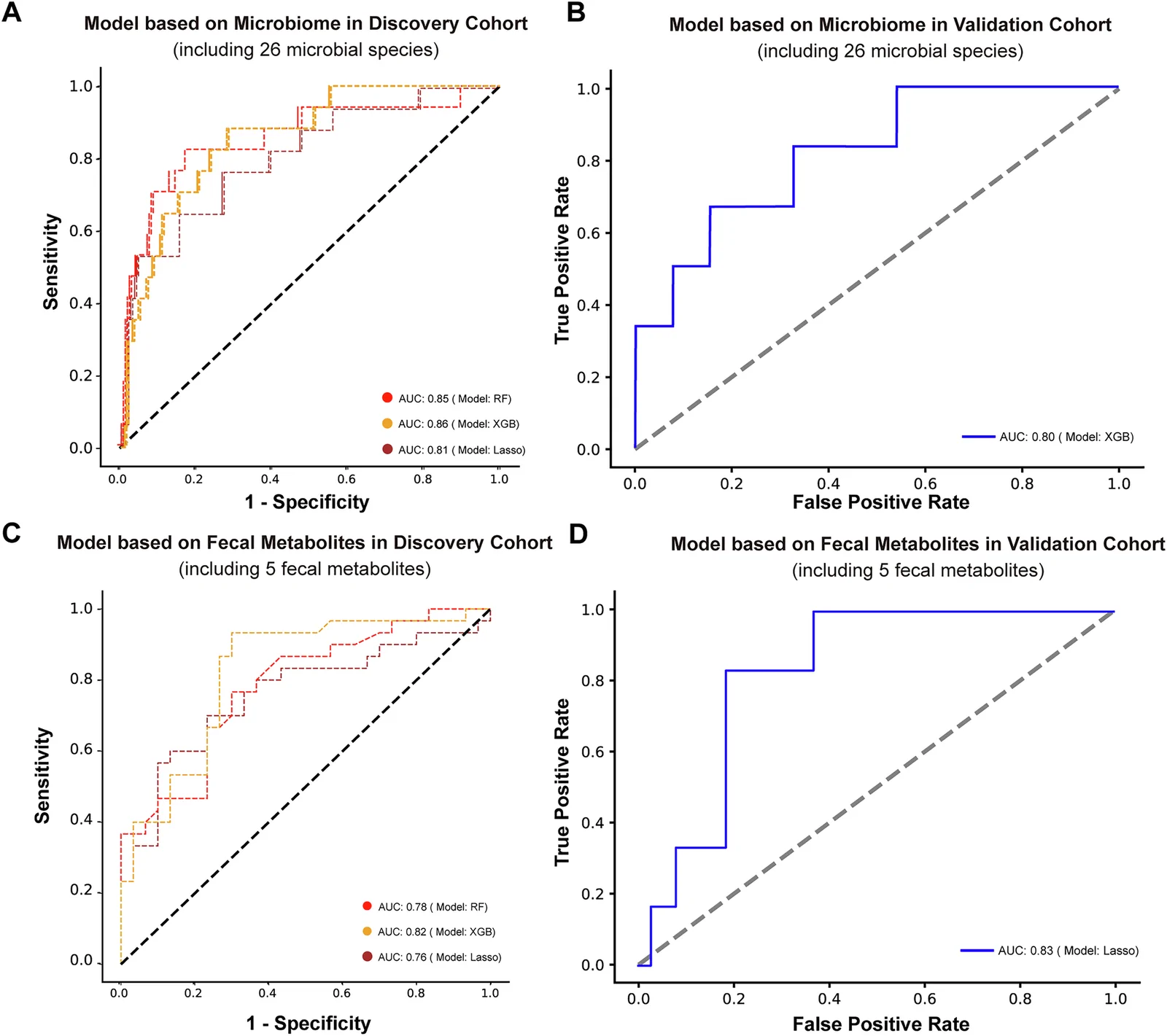
Performance of bacterial-fungal and metabolic biomarkers for diagnosis of postoperative infection. **A**,**B** The model based on microbial biomarkers for postoperative infection was constructed and achieved AUROC = 0.86, 0.85 and 0.81 in the discovery cohort and AUROC = 0.80 in the validation cohort. **C**,**D** The model based on fecal metabolites biomarkers for postoperative infection was constructed and achieved AUROC = 0.78, 0.82 and 0.76 in the discovery cohort and AUROC = 0.83 in the validation cohort.

To avoid the possible overfitting, we recruited a validation cohort by enrolling another 58 KT recipients (Table S3, S4) and performed metagenomic sequencing and metabolome profiling. The selected microbial (including 6 CAGs) and fecal metabolic features (N-acetylputrescine, hydroxyproline, indoleacetic acid, L-citrulline, and stachyose) were subsequently used for supervised machine learning to evaluate their diagnostic potential for post-transplant infection. Based on the biomarkers previously identified, we trained the model with key features of gut microbes and metabolites by using several different machine learning methods: Random Forest (RF), Extreme Gradient Boosting (XGB) and LASSO (Least Absolute Shrinkage and Selection Operator) Regression (Fig. 5A, C). The best area under receiver operating characteristic (AUROC) of model based on fungal-bacterial biomarkers achieved at 0.80 in validation cohort by XGB method (Fig. 5B). For microbiome-related gut metabolites, the best AUROC was 0.83 in validation cohort by LASSO method as well (Fig. 5D). Together, both gut microbial and metabolite features exhibited powerful diagnostic performance of postoperative infection.

## Discussion

Due to metabolic disorder as of renal insufficiency, the ESRD patients are greatly threatened by postoperative infection. Metabolites can act as mediators between the gut microbiome and host to regulate infection and improve prognosis outcomes^14,27,28^. With growing recognition of the importance of gut microbiome in transplantation, the intricate relationship among gut microbiome, its derived metabolites and host metabolism merits further investigations^6,7^. In our study, we established a prospective and longitudinal KT cohort from different centers and integrated multi-omics analysis to identify the high-accuracy diagnostic markers associated with postoperative infection. Our findings not only uncovered the postoperative infection-specific multi-kingdom microbial network dynamics, but also revealed the microbiome derived fecal metabolites with powerful diagnostic ability for postoperative infection in KT.

While previous studies have largely focused on bacterial microbiota, emerging evidence highlight the role of non-bacterial microbes in human diseases^29,30^. By multi-kingdom metagenomic analysis, we identified both bacterial and fungal species specific to postoperative infection. We identified the microbiome associated with postoperative infection which was classified into CAG 1-6. Importantly, the presence and viability of key fungal species were validated through culture-based approaches, providing potential biological evidence which complements with our metagenomic analysis. *Candida*, an opportunistic human fungal pathogen^30–32^, were more frequently observed in non-infected recipients in our study. Given the complex mechanisms of fungal-bacterial interactions, several conjectures have been made about this: The pathogenicity of *Candida* is dependent on morphological characteristics and virulence^33,34^. *Candida* sp. may reduce lung inflammation by inhibiting the amplification of pathogens^35^, and a mechanism that may warrant further exploration in the context of post-transplant infection. Phenylpyruvic acid (PPA) produced by *Candida* helps to enhance macrophage activity thereby attenuating sepsis^36^.

Inreasing evidence demonstrated that there existed dynamic interactions across the multi-kingdom microbial community^19,37^. We discovered gut metabolites associated with postoperative infection and the potential risk factors. In particular, we observed coordinated cross-kingdom associations among microbial communities, gut metabolites, and host features related with postoperative infection, suggesting a potential multi-layered association. We found that among the microbial communities positively associated with infection, *Enterobacter*-dominated CAG1 and *Citrobactor*-dominated CAG2 showed the strongest correlation with the gut metabolites pattern F2. *Enterobacter* and *Citrobacter* have been reported as key microbes responsible for the production of putrescine^38^. *Streptococcus*-dominated CAG4 was strongly associated with altered plasma metabolite profiles and may be linked to intestinal barrier dysfunction^21^ and host inflammation^39^. Among CAG4, *S. koreensis* was reported to be involved in the initiation and progression of colorectal cancer, suggesting their role in intestinal carcinogenesis^40^.

Increasing evidence has recognized the protective role of the gut microbiome against infection while postoperative treatment disrupts the microbiome, increasing susceptibility to pathogens^41,42^. Probiotics and postbiotics play pivotal roles in maintaining Intestinal homeostasis and host health^43^. We found that in infected recipients, CAG5 and CAG6 (*Akkermansia*, *Penicillium sp., Candida sp., Ruminiclostridium sp*. and etc.) were depleted. Decreased *Ruminiclostridium sp*. was reported to be associated with diarrhea post transplantation^44^. *Ruminiclostridium sp*. was shown to secrete an extracellular, multi-enzyme machinery cellulosome, which efficiently degrades cellulose^45^. Our study also discovered that *Ruminiclostridium papyrosolvens* and *Ruminiclostridium herbifermentans* were significantly positively correlated with Enterolactone. Also, fiber intake favoured the reduction of infections and was positively correlated with *Ruminiclostridium* and Enterolactone. It has been demonstrated that Enterolactone is a lignan produced by gut microbes on plant metabolism^22,23^. These observations support a putative association among fiber intake, *Ruminiclostridium* spp., and enterolactone, consistent with a potential diet-microbiome-metabolite interaction axis, although causal relationship remain undetermined.

Admittedly, our study also has some limitations. While our findings provide insights into postoperative infection-associated microbial and metabolic alterations in kidney transplant recipients, the generalizability of these observations to other infectious conditions or clinical settings awaits further investigation. In addition, due to the observational design of the present study, causal relationships between microbial and metabolic alterations and postoperative infection cannot be inferred. Future investigations aimed at addressing microbiome and metabolite imbalances are required to evaluate whether restoring specific bacterial species or their metabolites can help reduce the incidence of infection.

In conclusion, our study explored the multi-kingdom microbial network and highlighted the microbiome-metabolism crosstalk in microbe-host interaction. Insights into microbiome-metabolite interactions may inform future research on diagnostic and therapeutic strategies. The model, validated both internally and externally, offers preliminary clinical utility. Timely detection of gut microbiome and their metabolites may help prevent postoperative complications and support renal recovery.

## Methods

### Study population and design

This prospective cohort consisted of adult KT recipients recruited between April 2023 and April 2024 and followed up for 1 year. Recipients in discovery cohort were recruited from four transplantation centers in China, including Tianjin First Central Hospital (TJF), Tongji Hospital of Huazhong University (WHT), Shanghai General Hospital (SHP) and West China Hospital of Sichuan University (SCH). Recipients in validation cohort were recruited from two transplantation centers including First Affiliated Hospital of Zhengzhou University (ZUF) and West China Hospital of Sichuan University (SCH2). All procedures performed were in accordance with the ethical standards of the local ethics review committees at each recruiting center. This study was approved by the Tianjin First Central Hospital Medical Ethics Committee (2022N145KY), Tongji Hospital Medical Ethics Committee (TJ-IRB20221201), Shanghai General Hospital Medical Ethics Committee (2022KY113), West China Hospital Medical Ethics Committee (huaxi2024661), and The First Affiliated Hospital of Zhengzhou University Ethics Committee (2022-KY-1449-002). Written informed consent was obtained from all the recipients prior to their surgery, and all of donations were voluntary and altruistic in all cases, and were in accordance with the ethical guidelines of the Declaration of Helsinki.

Adult recipients (≥18 years of age) undergoing KT for the first time were considered eligible for enrollment. Exclusion criteria: (i) Patients experienced serious post-transplant complications (such as acute immune rejection). (ii) Patients failed to provide sufficient longitudinal sample. (iii) Patients actively undergoing treatment in an FMT clinical trial.

Based on follow-up observations, recipients were categorized into two groups: those who experienced infections and those who did not. Infection was defined as follows: the presence of clinical symptoms with pathogen detection. Infections were classified by diagnosis, anatomic location, pathogen, signs, and diagnostic studies, which can be found in the Table S1. Demographics, clinical and medicine data for different cohorts are available in Tables S2, 3, and 4.

### DNA extraction

Fecal and environmental samples were collected in sterile collection tubes and immediately stored at −80 °C until further processing. All samples (including fecal specimens, environmental swabs, and laboratory blank controls) were extracted centrally in a single laboratory using an identical workflow. Genomic DNA (gDNA) was extracted using the Fecal Genomic DNA Kit (catalog no. CW2092S; CWBIO) following the manufacturer’s protocol, with several modifications: protease inhibitor (10 mg/ml) and lysozyme (10 mg/ml) were added before grinding, and acid-washed glass beads (catalog no. G8772; Sigma-Aldrich) were incorporated. The samples were then processed with a FastPrep-24™ 5G homogenizer (MP Biomedicals, USA) for 40 s, followed by 1-min ice baths, repeated four times to enhance the extraction of fungal DNA. For each extraction batch, PBS extraction blank controls were processed in parallel under identical conditions to monitor reagent- and laboratory-level contamination.

### Shotgun metagenomic sequencing

Shotgun metagenomic sequencing was applied to fecal samples from transplant recipients. DNA quantification was conducted with a Qubit 3 fluorometer (Thermo Scientific), and agarose gel electrophoresis was carried out using a DYY-6C electrophoresis apparatus (Beijing Liuyi). Gel images were captured with the BG-gdsAUTO(130) gel imaging system (Beijing Baijing). Agarose for gel preparation was obtained from Invitrogen (catalog no. 75510-019), and the DNA size marker used was DL2000/DL15000 (Takara). For electrophoresis, TAE buffer (Invitrogen, catalog no. AM9870) was utilized. Libraries were sequenced on the Illumina NovaSeq 6000 platform using paired-end 150 bp reads, with a target output of 10 Gb per sample.

### ITS1 and 16S rDNA amplicon sequencing

To profile environmental and laboratory contamination, both 16S and ITS1 rDNA amplicon sequencing were applied to all negative control samples. Sequencing of the ITS1 and 16S was conducted on an Illumina NovaSeq PE250, and the sequencing data was processed using the QIIME2 analysis pipeline. For bacterial analysis, the V3-V4 hypervariable regions of the 16S gene were amplified using the widely used universal primers 338F (5′-ACTCCTACGGGAGGCAGCAG-3′) and 806R (5′-GGACTACHVGGGTWTCTAAT-3′), which target conserved region common to most bacterial phyla, ensuring broad taxonomic coverage. Fungal community profiling was performed by amplifying the ITS1 region using custom primers: Forward (F) primer ACACACCGCCCGTCGCTACT and Reverse (R) primer TTTCGCTGCGTTCTTCATCG, selected to specifically amplify fungal species while minimizing non-target amplification. The community composition for each sample was determined at various taxonomic levels (Kingdom, Phylum, Class, Order, Family, and Genus)^46^.

### RT-qPCR assay

Of the extracted total fecal DNA, 1 µL was used as a template for qPCR analysis using the FungiQuant probe and primers^47^ for fungal burden. The data were normalized by the initial wet weight and DNA concentration of the fecal samples.

### Fungal culturomic and single-colony identification from stool samples

Liquid stool samples (200 µL) were plated on Sabouraud dextrose agar plates supplemented with 0.01 mg/mL vancomycin, 0.1 mg/mL gentamicin, and 0.1 mg/mL amikacin to culture viable microorganisms at 37 °C for 72 h. Colony-forming units were calculated by counting single colonies. Monoclonal strains were subsequently analyzed by matrix-assisted laser desorption/ionization time-of-flight mass spectrometry (MALDI-TOF MS). Single colonies were transferred directly onto the MALDI target plate, overlaid first with 1 µL of formic acid, followed by 1 µL of matrix solution consisting of alpha-cyano-4-hydroxycinnamic acid (CHCA) dissolved in acetonitrile and trifluoroacetic acid. Then the sample was dried, and spectra were acquired using a MALDI-TOF mass spectrometer in the positive ion mode. These mass spectra were compared to reference spectra in the MALDI Biotyper database for species identification. Identification was based on the highest score obtained from the database search, with a score >2.0 considered reliable for species identification. Single colonies were then picked and stored in glycerol stocks at −80 °C.

### LC-MS analysis

Metabolites were extracted from samples that were slowly thawed at 4 °C and subsequently mixed with a precooled methanol/acetonitrile/water solution (2:2:1, v/v). The mixture was vortexed, sonicated at low temperature for 30 min, and incubated at −20 °C for 10 min. After centrifugation at 14,000 × *g* for 20 min, the supernatant was vacuum-dried. Chromatographic separation was performed using a Vanquish LC ultra-high-performance liquid chromatography (UHPLC) system equipped with a HILIC column, maintained at 25°C with a flow rate of 0.5 mL/min and an injection volume of 2 μL. The mobile phases consisted of water with 25 mM ammonium acetate and 25 mM ammonia (Phase A), and acetonitrile (Phase B). A gradient elution program was employed as follows: 0–0.5 min with 95% B, 0.5–7 min with B decreasing linearly from 95 to 65%, 7–8 min with B decreasing from 65 to 40%, 8–9 min with B maintained at 40%, and 9–9.1 min with B increasing linearly from 40 to 95%. The samples were stored at 4 °C in the autosampler. To reduce signal fluctuations, samples were analyzed in a randomized sequence, with QC samples inserted to monitor system stability and data reliability. Mass spectrometric analysis was conducted using an Orbitrap Exploris™ 480 mass spectrometer (Thermo Fisher Scientific) with electrospray ionization (ESI) in both positive and negative ion modes. Ion source parameters were as follows: nebulizer gas (Gas 1) at 50, auxiliary gas (Gas 2) at 2, ion source temperature at 350 °C, and spray voltages of 3500 V in positive ion mode and 2800 V in negative ion mode. The mass-to-charge ratio scan range was 70–1200 Da for both first- and second-order scans, with a resolution of 60,000 and an accumulation time of 100 ms per scan. Data were collected in data-dependent acquisition (DDA) mode with a dynamic exclusion time of 4 s, and a total of 10 fragment spectra were collected per scan.

### Metagenomic data processing

We processed and cleaned high-throughput sequencing data using KneadData (v0.12.0) to remove contaminants and low-quality sequences. Low-quality reads were automatically detected and removed using Fastp (v0.21.0). The remaining reads were mapped using Bowtie2 (v2.4.2) to the mammalian genomes (hg38, canFam4, mm10, and galGal4; UCSC Genome Browser), 81,152 bacterial plasmids (NCBI RefSeq database accessed in April 2024), 11,515 complete plastids (NCBI RefSeq database accessed in April 2024), an additional UniVec database (NCBI RefSeq database accessed in April 2024), and the CHM13 human reference genome (NCBI RefSeq database accessed in July 2024). We employed Kraken2 (v2.1.3) to assign taxonomy to the metagenomic reads for bacteria, archaea, and fungi. Kraken2 is an advanced metagenomic classifier that utilizes a k-mer-based algorithm. A custom database was built, comprising 64,570 bacterial, 2024 archaea, and 14,993 viral reference genomes from the NCBI RefSeq database (accessed in April 2024), as well as 14,543 fungal reference genomes from the NCBI RefSeq, GenBank, FungiDB (http://fungidb.org), and Ensembl (http://fungi.ensembl.org) databases (accessed in April 2024). The database was generated using Jellyfish (v2.3.0) by computing distinct 31-mers across the reference library. Each k-mer from the reads was mapped to the lowest common ancestor of all reference genomes to ensure accurate matching. Subsequently, each query was classified to the specific taxonomic unit with the highest total k-mer hit rate by trimming the general classification tree linked to the mapped genomes. To estimate taxonomic abundance more accurately, Bracken (v2.5.0) was employed.

### Decontamination analysis

To remove potential contaminants, we performed the decontam R package (https://github.com/benjjneb/decontam)^48^ with the prevalence-based method (threshold = 0.1). For each clinical center, the corresponding seven environmental samples (swabs from operating room equipment, operating room air, surgical beds, and air samples from the four cardinal directions of patient wards) were used as center-specific negative controls to identify and remove environmentally derived contaminant taxa from that center’s metagenomic features. Subsequently, three PBS extraction blank controls, which had been processed through the whole extraction and library preparation workflow alongside fecal samples, were applied as laboratory-level negative controls across all transplantation centers to remove reagent-derived contaminants. The decontaminated features from each center were merged for subsequent analyses.

### Batch effect correction

For the ZUF center and the SCH2 center, microbial abundance matrices from two independent batches were merged and subjected to total-sum scaling normalization. Batch effects were corrected using MMUPHin^49^ with batch specified as the adjustment variable, while preserving the biological effect of infection status (Group) as a protected covariate. Corrected abundance matrices were subsequently used for downstream modeling analyses.

### Metagenomic statistical analysis

We compared infected and non-infected recipients using Multivariate Association with Linear Models 2 (MaAsLin2). with age, sex, and time point as fixed effects, while individual and center included as a random effect to account for repeated longitudinal sampling and potential inter-center heterogeneity, respectively. To ensure the stability of infection-associated microbial features across imbalanced groups, we performed bootstrap-based downsampling of the non-infected group at each timepoint (T1, T2, and T3). For each iteration (*n* = 100), we randomly sampled a subset of non-infected recipients matching the number of infected cases, and conducted differential abundance testing using MaAsLin2^50^, with fixed effects for group, age, and sex and random effects for clinical centers.

### Microbial gene functional annotation and KEGG pathway analysis

Microbial gene functional annotations were performed using eggNOG-Mapper v2.0 (https://github.com/eggnogdb/eggnog-mapper), with functional categories derived from EggNOG and KEGG pathways. High-quality reads were first preprocessed and assembled into contigs using Megahit v1.2.9, with contigs shorter than 500 bp excluded from further analysis. Gene prediction was conducted using Prodigal v2.6.3 in metagenome mode. Functional annotations were assigned to the reference using EggNOG Mapper v2.0.1, based on EggNOG orthology data. The relative abundances of EggNOG genes, KEGG KO groups, or pathways were calculated by summing the relative abundances of genes assigned to the same KOs or pathways. To identify differentially abundant microbial genes associated with infection and transplantation status, differential expression analysis was performed using DESeq2^51^, applied to infected versus non-infected recipients. Genes with Benjamini-Hochberg-adjusted *p*-value (padj) <0.05 were considered significantly differentially abundant. KEGG KO identifiers of significant genes were extracted and submitted to enrichment analysis using the enrichKEGG function in clusterProfiler^52^, with organism set to “ko”, *p*-value cutoff of 0.05, and *q*-value cutoff of 0.2.

### Metabolomic statistical analysis

Raw data were processed using XCMS software for peak alignment, retention time correction, and peak area extraction. The extracted features from XCMS underwent metabolite identification and preprocessing, which included the removal of ions with more than 50% missing values, imputation of missing values via KNN (k-nearest neighbors) imputation, and the exclusion of features with a relative standard deviation (RSD) greater than 50%. The quality of the processed data was then assessed through six quality control metrics, including comparing the total ion chromatogram (TIC) of QC samples through spectral overlay, conducting principal component analysis (PCA) of the overall samples, evaluating the correlation of QC samples, performing the Hotelling’s T2 test on the overall samples, constructing a multivariate control chart (MCC), and calculating the RSD of QC samples. Considering that metabolites was sequenced in two batches, we corrected the batch effect by linear regression based on bypass samples (samples tested in both batches, see metadata). Non-parametric statistical tests were used for differential comparison. Spearman’s rank correlation analysis was performed to evaluate monotonic associations among microbial species and metabolites. Prior to correlation analysis, metabolite concentrations were log-transformed to reduce skewness and improve distributional stability. Correlation coefficients and corresponding *p* values were calculated using two-sided *t*-test.

### Feature selection

Fecal microbial biomarkers: Infection-associated microbial species were first identified by MaAsLin2 analyses, including postoperative alterations relative to pre-transplantation and differential abundance between infected and non-infected recipients. These species were subsequently clustered into CAGs using Louvain modularity analysis. Within each CAG, species ranked among the top three by absolute MaAsLin2 coefficient in at least one comparison were selected as candidate microbial biomarkers, yielding 26 microbial features across 6 CAGs.

Fecal metabolite biomarkers: Candidate fecal metabolites were selected based on the following criteria: (1) significant differential abundance between infected and non-infected recipients (*p* < 0.05 and |log_2_ fold change| > 1); (2) consistent directional changes across postoperative time points; (3) biological annotation supported by MetaBio and PubChem databases; and (4) significant associations with circulating plasma metabolites. Five fecal metabolites were retained for downstream modeling analysis.

### Model development and validation

After feature selection, 26 microbial species representing six CAGs and 5 fecal metabolites linked to host responses were included in model construction. The discovery cohort was randomly split into training and testing sets at an 8:2 ratio, and a grouped 10-fold cross-validation framework was implemented, with patient Individual ID specified as the grouping variable and infection status as the stratification variable to prevent data leakage arising from repeated longitudinal sampling. Within each fold, class imbalance between infected and non-infected samples was addressed by random downsampling of the majority class to achieve a 1:1 ratio. Three machine learning models, including RF, XGB, and LASSO Regression were were evaluated. The final model was subsequently applied to the independent external validation cohort comprising 58 KT recipients for assessment of generalizability. Model performance was assessed by the area under the receiver operating characteristic curve (AUROC) in both internal cross-validation and the independent external validation cohort.

## Supplementary information

Supplementary Information

## Data Availability

All data used for the analysis are availabl through public repositories. The metagenomic sequencing data is available on NCBI Bioproject (PRJNA1223318) and National Omics Data Encyclopedia (OEP00006065, OEP00007081 and OEP00007082). Fecal metabolomic data and Plasma metabolomic data have also been deposited at National Omics Data Encyclopedia and are publicly available under OEP00006043 and OEP00006044 as of the date of publication.
