# Supplementary material for: Longitudinal multi-omics analysis identify multi-kingdom microbiome-host interaction dynamics and diagnostic biomarkers of postoperative infection after kidney transplantation
Source: NPJ Biofilms Microbiomes. 2026 Jul 20;12:230. doi: 10.1038/s41522-026-01099-0 (PMC13751624; doi:10.1038/s41522-026-01099-0)
Supplement: Supplementary file 1 [file 41522_2026_1099_MOESM1_ESM.pdf]

Supplementary Figures

Longitudinal multi-omics analysis identify fungal-bacterial communities and metabolic biomarkers of postoperative infection in kidney transplantation recipients

Yun-Xuan Guan[1#], Lu Wang[1#], Ling-Xiang Kong[2#], Yan-Yan Sun[1], Jin-An Zhou[1], Xiu-Li Wang[1], Yong-Jing Ma[1], Li-Ping Guo[3], Tu-Run Song[2], Lan Zhu[4], Si-Jun Wang[4], Ya-Hui Huang[4], Ruo-Lin Tao[5,6], Wen-Jun Shang[5], Wen-Zhi Guo[5,6], Hui Wang[1*], Jia-Yin Yang[2*], Lin Zhong[8*], Gang Chen[4*], Jie Zhao[3,7*], Ning-Ning Liu[1*]

#These authors contributed equally.

*Corresponding authors. Email: Ning-Ning Liu, liuningning@shsmu.edu.cn; Jie Zhao, zjsecond@aliyun.com; Gang Chen, gchen@tjh.tjmu.edu.cn; Lin Zhong, zhonglin1@medmail.com.cn; Jia-Yin Yang, doctoryjy@scu.edu.cn; Hui Wang, huiwang@shsmu.edu.cn.

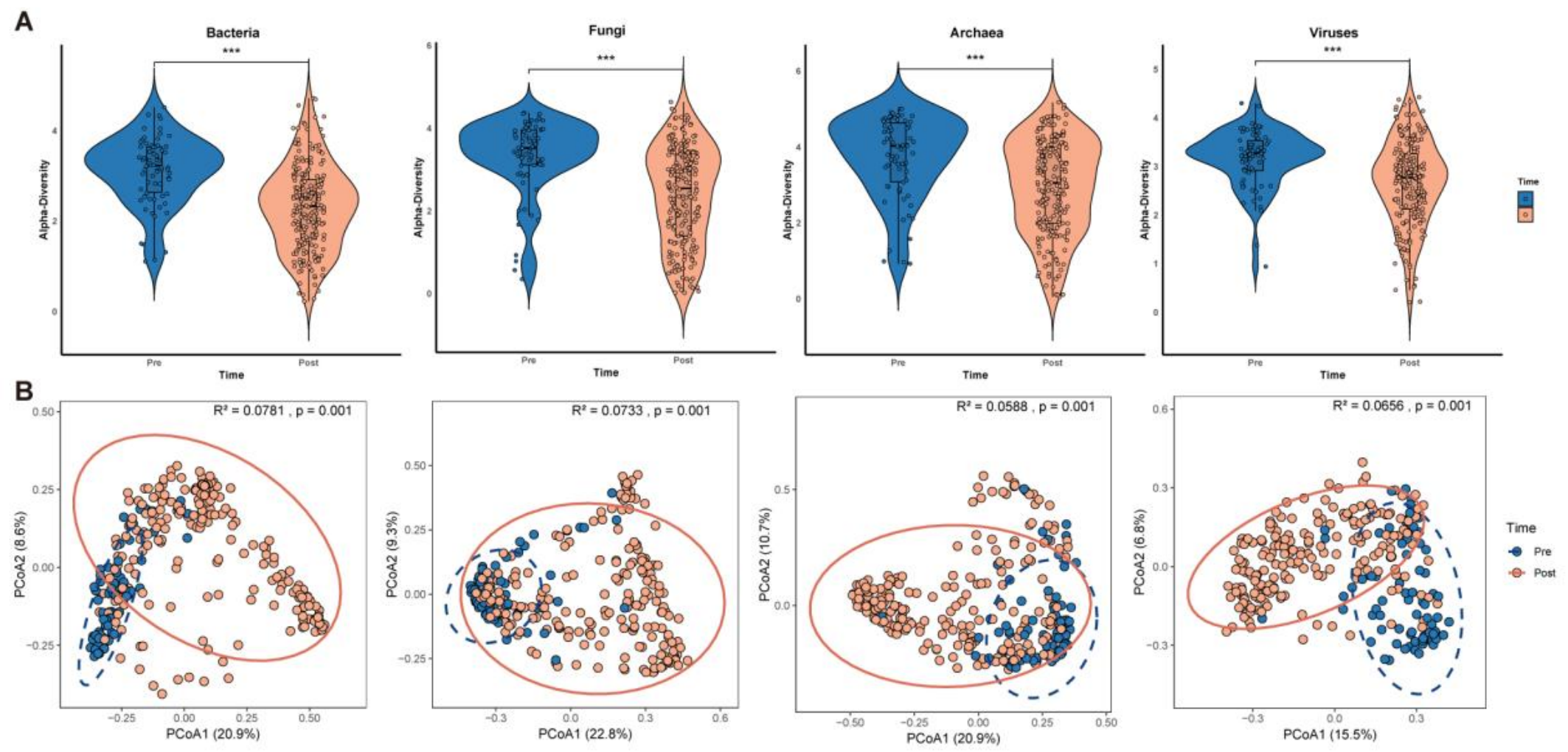

Figure S1, related to Figure 1. The alpha and beta diversity of the four-kingdom microbiome pre- and post-KT.

(A) Alpha-diversity measured by Shannon index of the four kingdom microbiome in recipients received kidney transplantation (orange, n=210) and pre-transplantation (blue, n=70).

(B) Principal coordinate analysis (PCoA) of samples based on Bray-Curtis distance, which shows that the four kingdom microbial composition was totally different between pre- and postoperative groups ($p = 0.001$).

Data are presented as mean ± SEM. Statistical significance (ns: not significant, *: $p < 0.05$; **: $p < 0.01$; ***: $p < 0.001$) were determined by unpaired two-tailed Student’s t test (A), permutation multivariate analysis of variance (B).

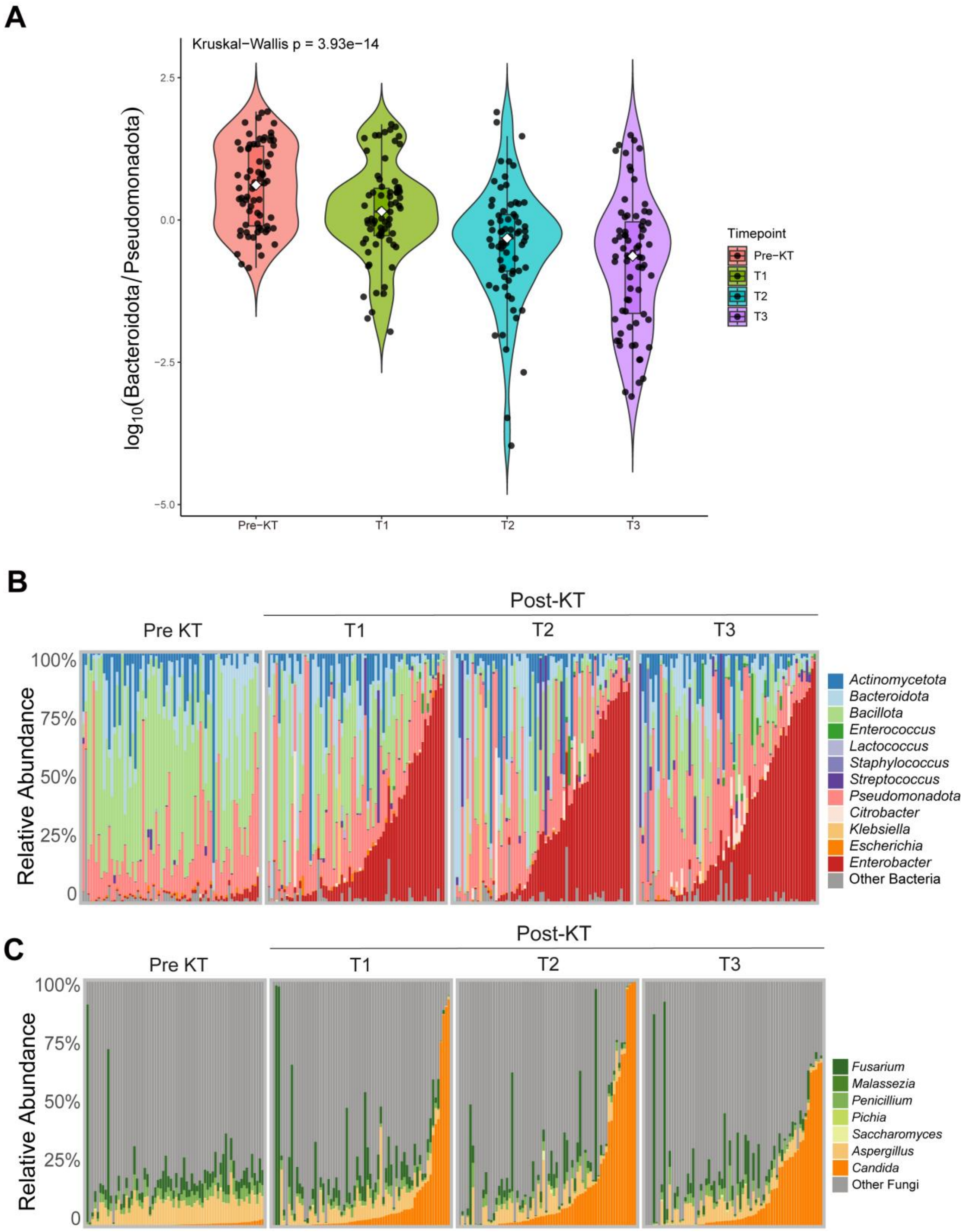

Figure S2, related to Figure 1. The longitudinal dynamic changes in the relative abundance of the bacteria pre- and post-KT.

(A) Bacterial community profiling based on relative abundance revealed a progressive decline in the *Bacteroidota/Pseudomonadota* ratio over time, indicating a temporal shift in community structure (Kruskal–Wallis test, $P = 3.93\times10^{-14}$).

(B-C) Stacked bar plots displayed the relative abundance of bacterial (B) and fungal (C) genera. Each bar represents an individual sample, with colors indicating specific genera as shown in the legend. Samples were grouped by time points to illustrate temporal trends in microbiome dynamics.

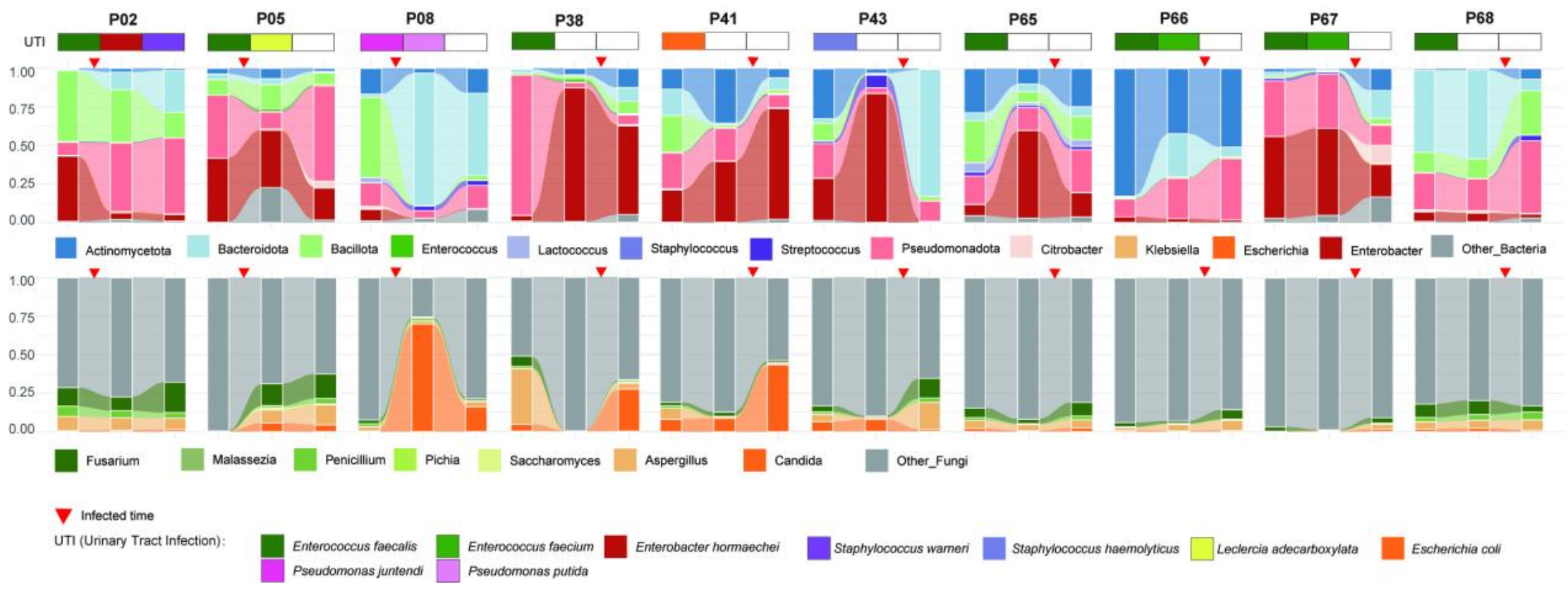

Figure S3, related to Figure 2. Temporal dynamics of bacterial and fungal abundance in infected recipients. Alluvial plots depict genus-level compositional shifts in the gut bacterial and fungal communities across T1, T2, T3 in infected patients. Each panel represents an individual recipient, and stream width reflects the relative abundance of each genus over time.

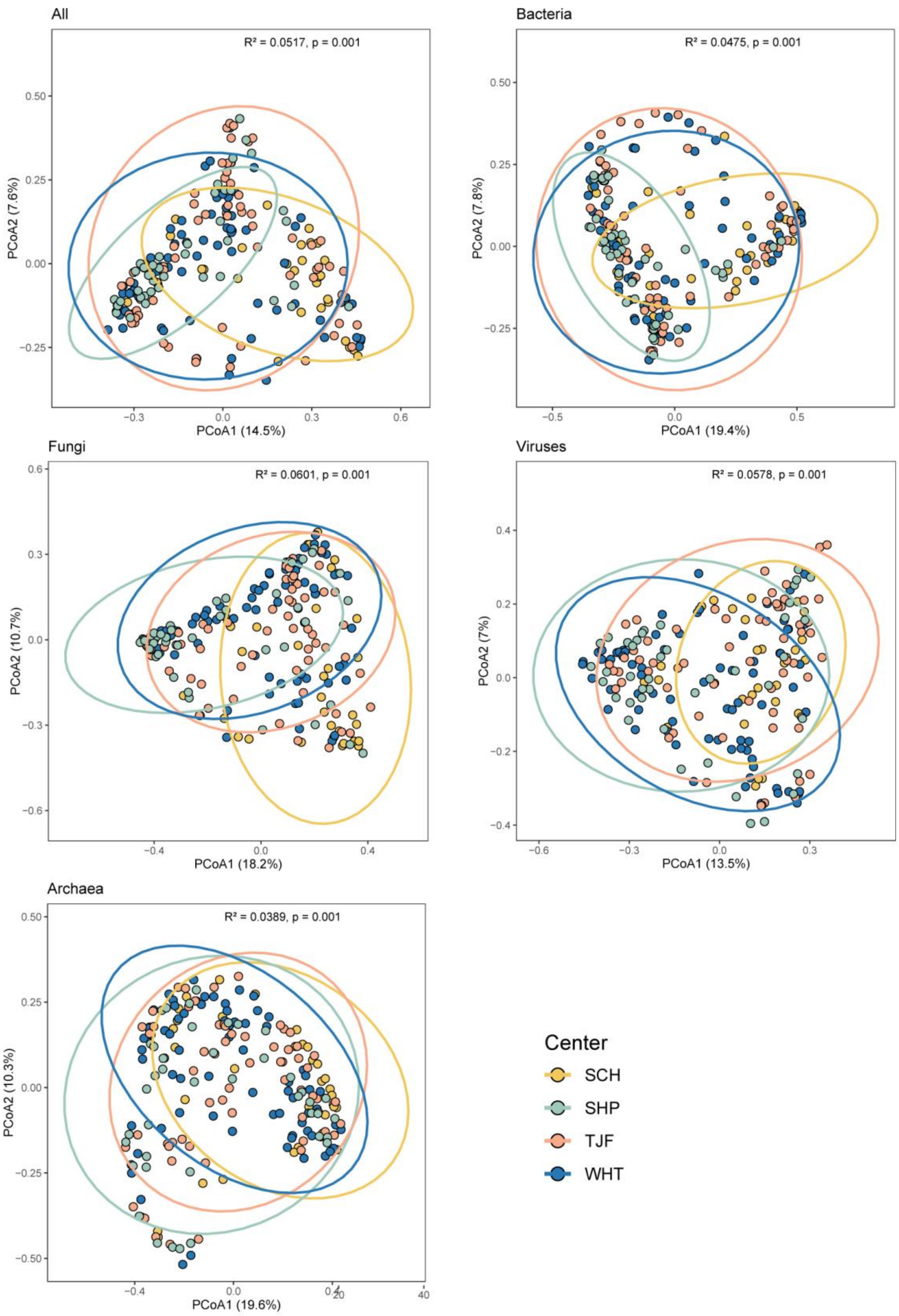

Figure S4, related to Figure 2. Assessment of inter-center batch effects in the discovery cohort. Principal coordinate analysis (PCoA) based on Bray-Curtis dissimilarity of metagenomic profiles from samples across the four discovery cohort centers.

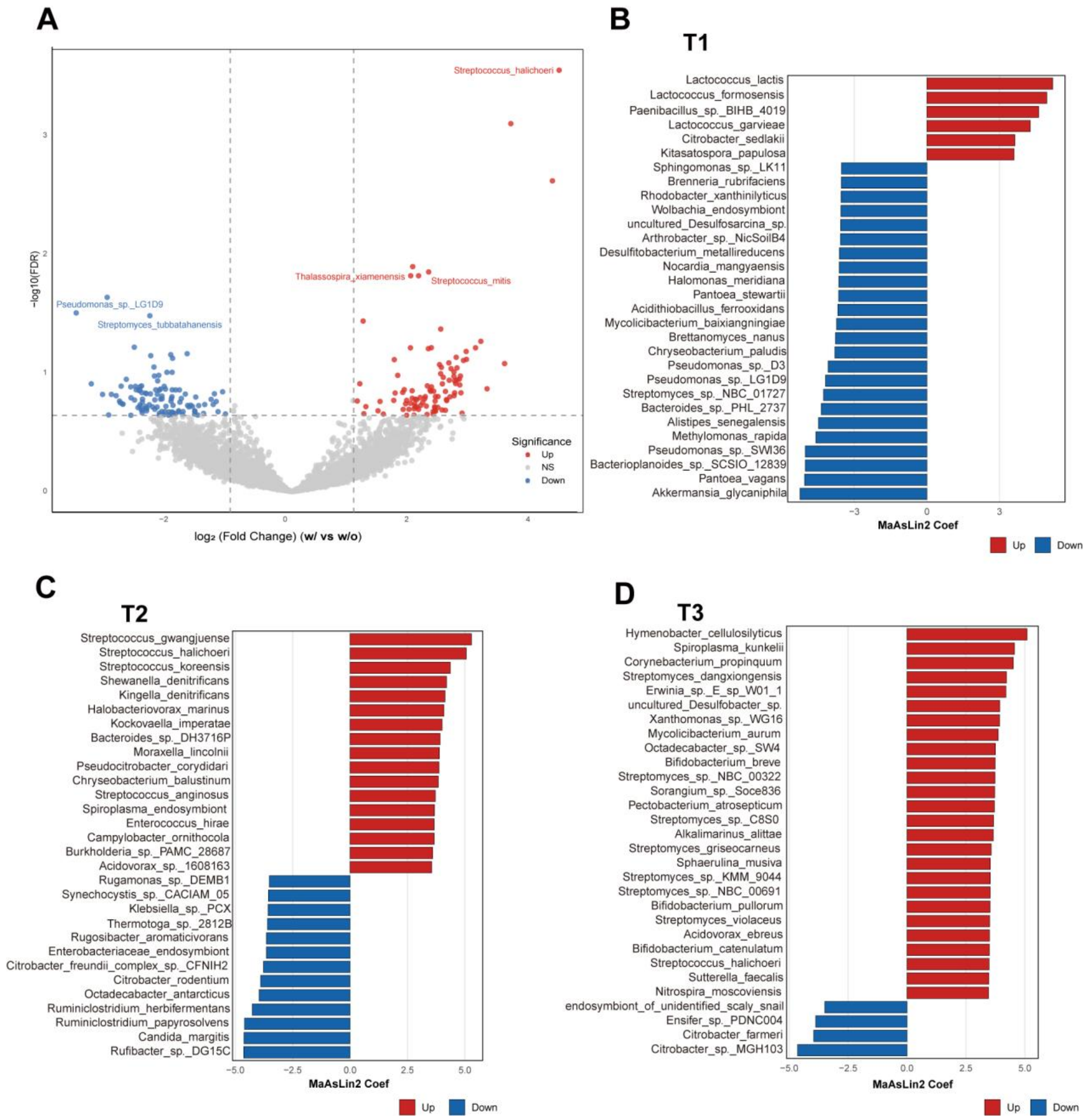

Figure S5, related to Figure 2. Differential microbial signatures in infected vs. non-infected recipients.

(A) Volcano plot showed differentially abundant microbial species between infected recipients (w/) and non-infected recipients (w/o). MaAsLin2 was applied to log-transformed, total sum scaled (TSS) microbial abundance data with fixed effects for infection status (Group), age, and sex, and a random effect for subject ID (Individual). Each dot represents one microbial species, with the x-axis showing the $\log_2$(Fold change) and the y-axis showing the -log10(FDR). Vertical and horizontal dashed lines indicate thresholds for significance ($|\log2FC|\geq1$ and $FDR<0.25$). Differentially enriched taxa in the infected group are colored in red, while depleted taxa are in blue; gray points denote

non-significant (NS) species.

(B-D) The bar plot represents the coefficients (coef) of the top significant differences microbes (FDR < 0.1) identified through MaAsLin2 analysis, between infection and non-infection groups at T1, T2 and T3.

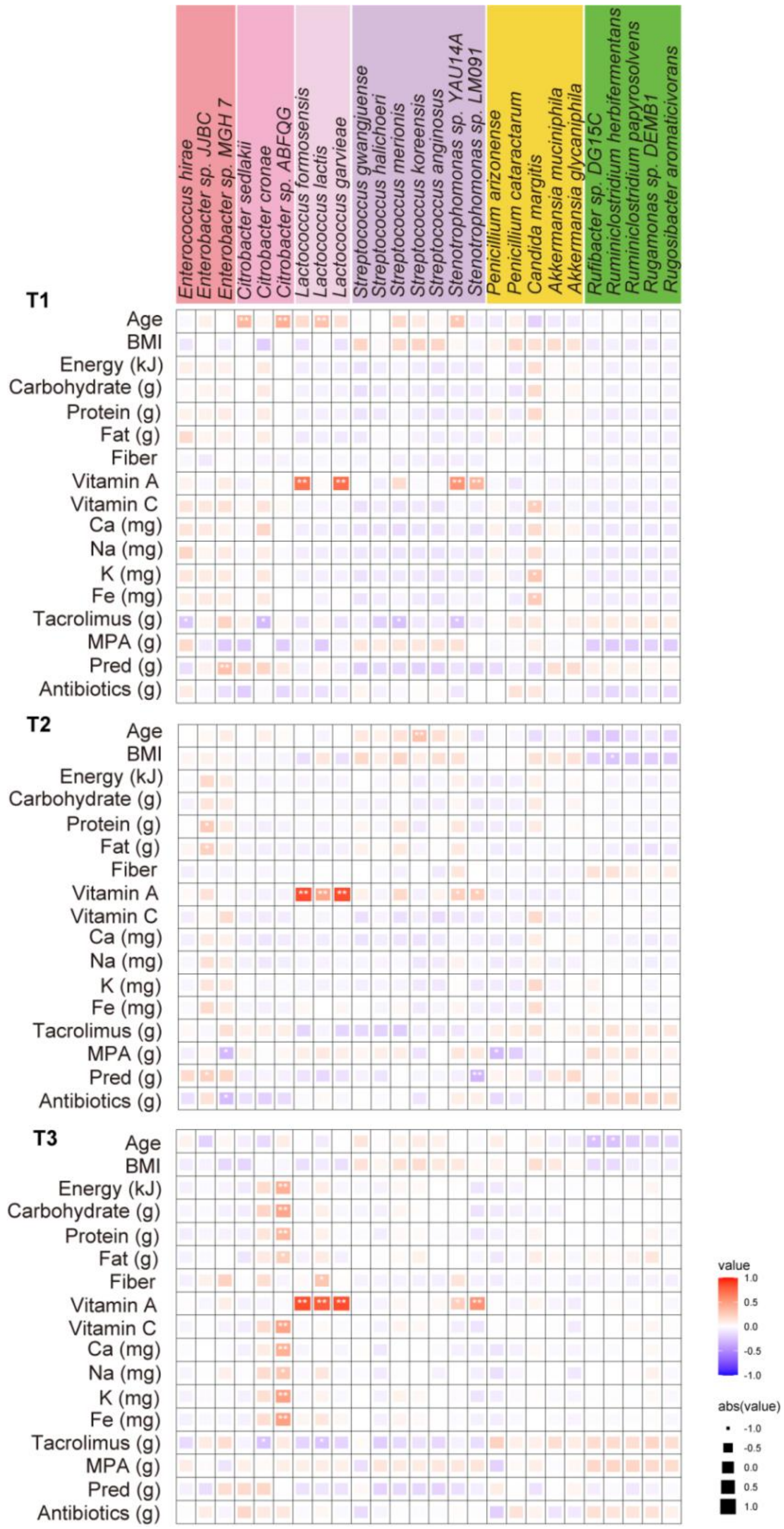

Figure S6, related to Figure 2. Other factors associated with gut microbiome dysbiosis in KT recipients. Correlation heatmap between demographic, lifestyle, and clinical covariates with microbiome at T1 ,T2 and T3. Data were presented as correlation coefficient. Statistical significance (ns: not significant, *: p < 0.05; **: p < 0.01; ***: p < 0.001) were determined by Spearman rank correlation analysis.

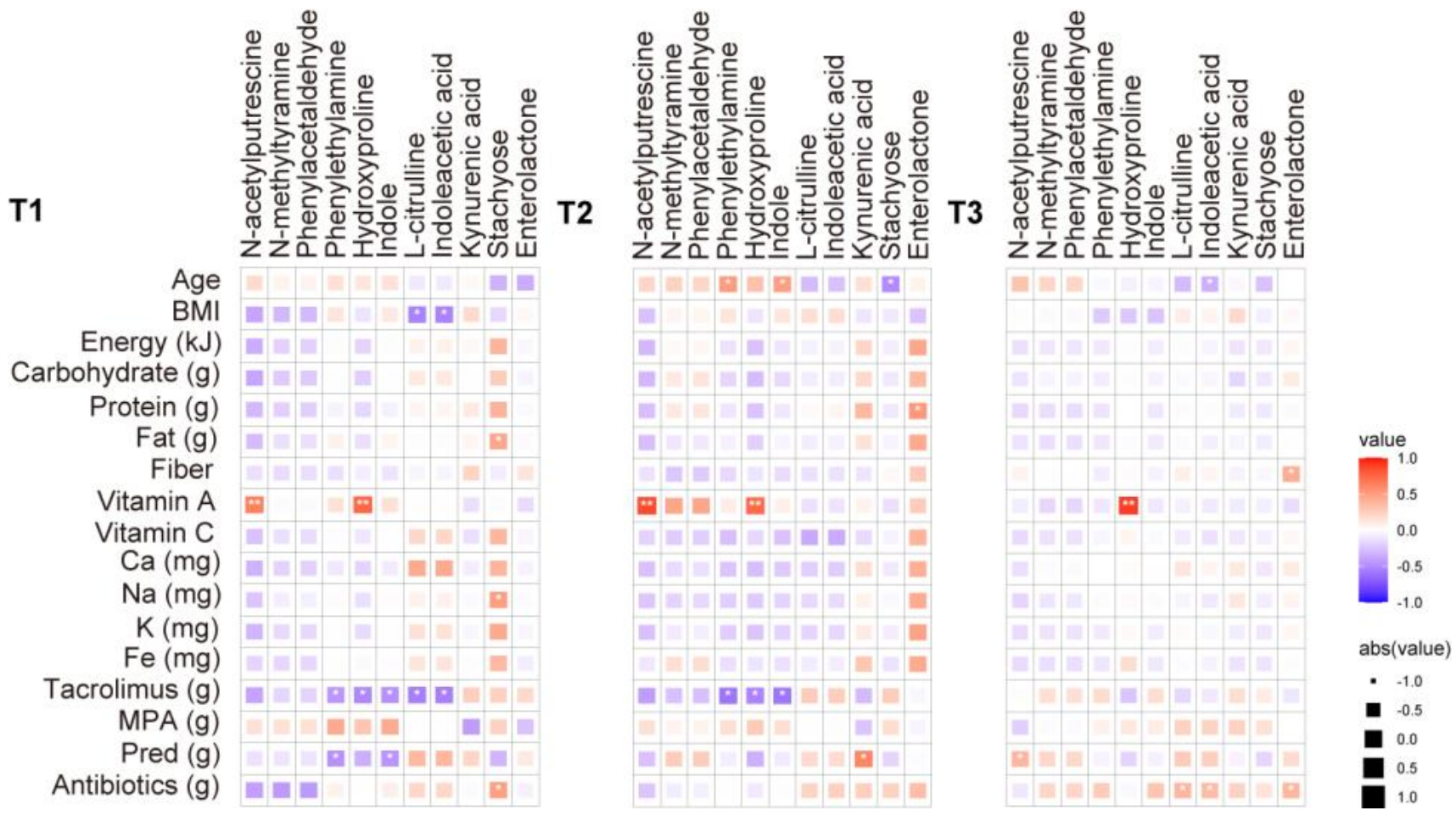

Figure S7, related to Figure 3. Other factors associated with gut metabolites in KT recipients. Correlation heatmap between demographic, lifestyle, and clinical covariates with gut metabolites at T1 ,T2 and T3. Data were presented as correlation coefficient. Statistical significance (ns: not significant, *: *p* < 0.05; **: *p* < 0.01; ***: *p* < 0.001) were determined by Spearman rank correlation analysis.

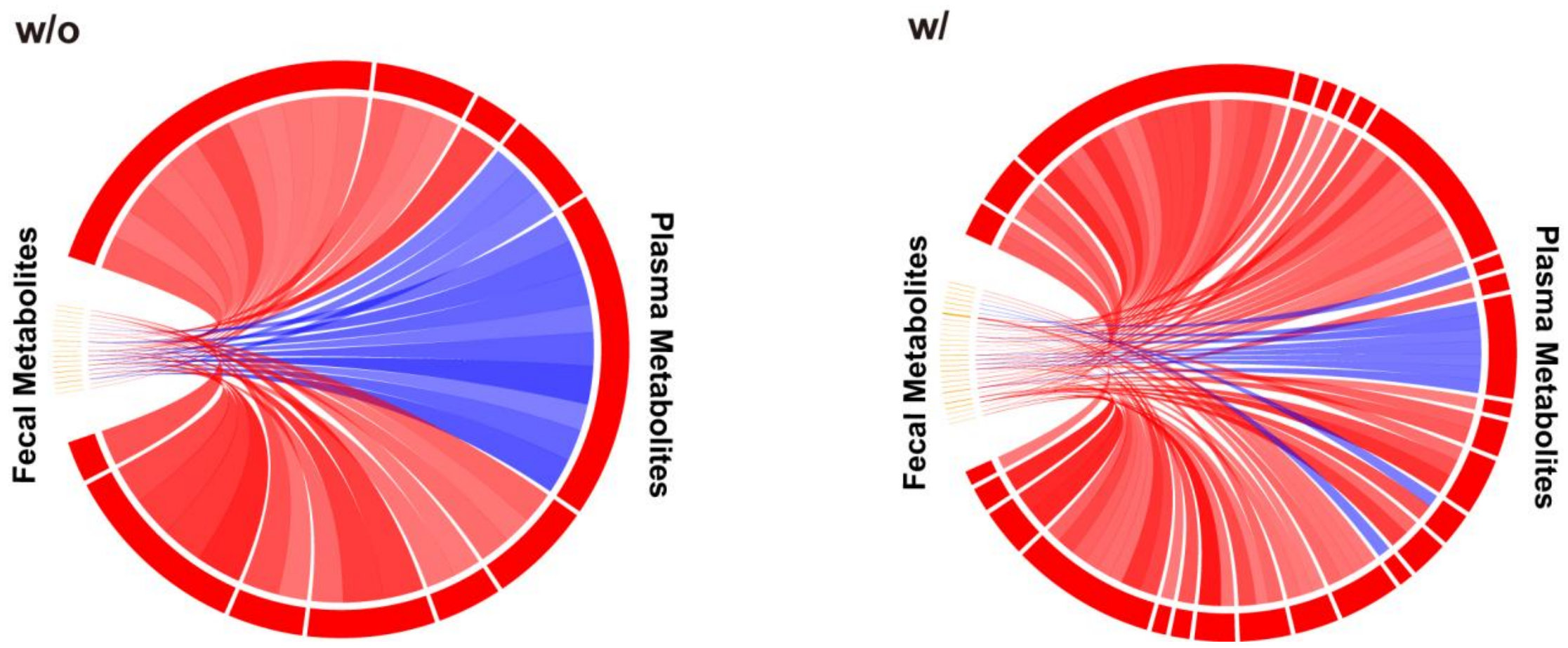

Figure S8, related to Figure 4. The correlation between gut metabolites and host metabolites of KT recipients with postoperative infection.

The chord diagrams present correlations between plasma and gut metabolites in non-infected recipients (left) and infected recipients (right). Plasma metabolites are colored in red, while gut metabolites are colored in orange. The correlation between metabolites is indicated by the color intensity of the links: red for positive correlations and blue for negative correlations, with the transparency of the color representing the magnitude of the correlation.

Table S1. Microbial and clinical characteristics of infections.

This table includes pathogens and infection sites identified through clinical diagnostic tests. Abbreviations: CMV: Cytomegalovirus; PCP: Pneumocystis pneumonia; COVID-19: Coronavirus Disease 2019.

Patient ID	Individuals	Site	pathogenic microorganism
**Patient02**	**SY004**	**Urinary tract infection**	***Enterobacter hormaechei***
**Patient02**	**SY004**	**Urinary tract infection**	***Enterococcus faecalis***
**Patient02**	**SY004**	**Urinary tract infection**	***Staphylococcus warneri***
Patient05	SY011		CMV
Patient05	SY011	Respiratory tract infection	PCP
**Patient05**	**SY011**	**Urinary tract infection**	***Enterococcus faecalis***
**Patient05**	**SY011**	**Urinary tract infection**	***Leclercia adecarboxylata***
Patient08	SY016		CMV
**Patient08**	**SY016**	**Urinary tract infection**	***Pseudomonas juntendi***
**Patient08**	**SY016**	**Urinary tract infection**	***Pseudomonas putida***
Patient38	WH057	Perinnephritis	*Klebsiella pneumoniae*
Patient38	WH057	Perinnephritis	*Enterococcus faecium*
**Patient38**	**WH057**	**Urinary tract infection**	***Enterococcus faecium***
Patient41	TJ304		CMV
Patient41	TJ304	Respiratory tract infection	COVID-19
**Patient41**	**TJ304**	**Urinary tract infection**	***Escherichia coli***
Patient41	TJ304	Perinnephritis	*Staphylococcus epidermidis*
**Patient43**	**TJ309**	**Urinary tract infection**	***Staphylococcus haemolyticus***
**Patient65**	**HX008**	**Urinary tract infection**	***Enterococcus faecium***
**Patient66**	**HX009**	**Urinary tract infection**	***Enterococcus faecalis***
**Patient66**	**HX009**	**Urinary tract infection**	***Enterococcus faecium***
Patient67	HX040	Respiratory tract infection	COVID-19
Patient67	HX040	Perinnephritis	*Staphylococcus epidermidis*
**Patient67**	**HX040**	**Urinary tract infection**	***Enterococcus faecalis***
**Patient67**	**HX040**	**Urinary tract infection**	***Enterococcus faecium***
Patient68	HX045	Respiratory tract infection	COVID-19
**Patient68**	**HX045**	**Urinary tract infection**	***Enterococcus faecalis***

Table S2. Demographic and clinical characteristics of non-infected and infected recipients

Variables represented as number and percent or median and interquartile range. Abbreviations: BMI: Body Mass Index; SBP: Systolic Blood Pressure; DBP: Diastolic Blood Pressure; PPI: proton pump inhibitors; MPA: Mycophenolic Acid; Pred: Prednisone.

	Infection (N=10)	Non-infection (N=60)	*p*
**Demographics**			
**Female (%)**	1 (10%)	28 (47%)	**0.029**
**Age (years)**	41.50 (36.00, 46.00)	36.50 (33.00, 47.00)	0.3
**Clinical data**			
**BMI (kg/m²)**	20.42 (19.53, 24.57)	21.58 (20.27, 24.05)	0.6
**SBP**	139.50 (132.00, 142.00)	136.00 (128.00, 146.50)	0.4
**DBP**	94.50 (82.00, 109.00)	90.00 (80.50, 101.00)	0.4
**PPI (%)**			0.5
**Omeprazole**	8 (80%)	41 (68%)	
**Rabeprazole**	2 (20%)	19 (32%)	
**Immunosuppressor (%)**			0.2
**Tacrolimus+MPA+Pred**	5 (50%)	46 (77%)	
**Tacrolimus+Pred**	3 (30%)	11 (18%)	
**Tacrolimus+MPA**	2 (20%)	3 (5%)	
**Antibiotics (%)**			0.7
**Cefoperazone**	5 (50%)	24 (40%)	
**Cefmetazole**	2 (20%)	11 (18%)	
**Piperacillin**	0 (0%)	11 (18%)	
**Carbapenems**	2 (20%)	10 (17%)	
**β-lactams+antifungals**	1 (10%)	4 (7%)	

Table S3. Demographic and clinical characteristics of Discovery cohorts and Validation Cohorts
Variables represented as number and percent or median and interquartile range. Abbreviations: BMI: Body Mass Index; SBP: Systolic Blood Pressure; DBP: Diastolic Blood Pressure; PPI: proton pump inhibitors; MPA: Mycophenolic Acid; Pred: Prednisone.

	Discovery (N=70)	Validation (N=58)	*p*
**Demographics**			
**Female (%)**	29 (41%)	19 (33%)	0.3
**Age (years)**	37.50 (33.00, 47.00)	42.00 (33.00, 50.00)	0.2
**Clinical data**			
**BMI (kg/m²)**	21.51 (20.20, 24.06)	21.50 (20.02, 23.40)	0.7
**SBP**	136.00 (128.00, 145.00)	135.00 (130.00, 145.00)	0.5
**DBP**	90.00 (82.00, 101.00)	86.50 (83.00, 93.00)	0.2
**PPI (%)**			**<0.001**
**Omeprazole**	49 (70%)	22 (38%)	
**Rabeprazole**	21 (30%)	0 (0%)	
**Ilaprazole**	0 (0%)	14 (24%)	
**Pantoprazole**	0 (0%)	6 (10%)	
**N/A**	0 (0%)	16 (28%)	
**Immunosuppressor (%)**			**<0.001**
**Tacrolimus+MPA+Pred**	51 (73%)	30 (52%)	
**Tacrolimus+Pred**	14 (20%)	0 (0%)	
**Tacrolimus+MPA**	5 (7.1%)	28 (48%)	
**Antibiotics (%)**			**<0.001**
**Cefoperazone**	29 (41%)	4 (6.9%)	
**Cefmetazole**	13 (19%)	15 (26%)	
**Piperacillin**	11 (16%)	8 (14%)	
**Carbapenems**	12 (17%)	3 (5.2%)	
**β-lactams+antifungals**	5 (7.1%)	28 (48%)	

Table S4. Demographic and clinical characteristics of non-infected and infected recipients in Validation Cohorts
Variables represented as number and percent or median and interquartile range. Abbreviations: BMI: Body Mass Index; SBP: Systolic Blood Pressure; DBP: Diastolic Blood Pressure; PPI: proton pump inhibitors; MPA: Mycophenolic Acid; Pred: Prednisone.

	Infection (N=6)	Non-infection (N=52)	*p*
**Demographics**			
**Female (%)**	4 (67%)	15 (29%)	0.062
**Age (years)**	36.00 (32.00, 49.00)	42.50 (33.00, 50.00)	0.5
**Clinical data**			
**BMI (kg/m²)**	20.20 (19.50, 26.50)	21.62 (20.15, 23.36)	0.6
**SBP**	136.00 (132.00, 150.00)	135.00 (129.00, 145.00)	0.5
**DBP**	87.00 (84.00, 96.00)	86.50 (83.00, 92.50)	0.7
**PPI (%)**			0.2
**Omeprazole**	2 (33%)	20 (38%)	
**Pantoprazole**	2 (33%)	4 (7.7%)	
**Ilaprazole**	0 (0%)	14 (27%)	
**N/A**	2 (33%)	14 (27%)	
**Immunosuppressor (%)**			0.4
**Tacrolimus+MPA+Pred**	4 (67%)	26 (50%)	
**Tacrolimus+MPA**	2 (33%)	26 (50%)	
**Antibiotics (%)**			>0.9
**Cefoperazone**	0 (0%)	4 (7.7%)	
**Cefmetazole**	2 (33%)	13 (25%)	
**Piperacillin**	1 (17%)	7 (13%)	
**Carbapenems**	0 (0%)	3 (5.8%)	
**β-lactams+antifungals**	3 (50%)	25 (48%)	
